# Data Resource Profile: The Australian Orthopaedic Association National Joint Replacement Registry (AOANJRR)

**DOI:** 10.1093/ije/dyaf078

**Published:** 2025-06-16

**Authors:** Yushy Zhou, Christopher J Wall, Jarrad Stevens, Andrew Fraval, Peter L Lewis, Michael J McAuliffe, Christopher J Vertullo, David R J Gill, James D Stoney, Durga Bastiras, Sophie Corfield, Bec Harvey, Michelle F Lorimer, Kathy Hill, Paul N Smith

**Affiliations:** Department of Orthopaedic Surgery, St Vincent’s Hospital Melbourne, Melbourne, Victoria, Australia; Department of Surgery, The University of Melbourne, Melbourne, Victoria, Australia; Australian Orthopaedic Association National Joint Replacement Registry (AOANJRR), South Australian Health and Medical Research Institute (SAHMRI), Adelaide, South Australia, Australia; School of Medicine, Rural Clinical School, University of Queensland, Toowoomba, Queensland, Australia; Department of Orthopaedic Surgery, St Vincent’s Hospital Melbourne, Melbourne, Victoria, Australia; Department of Orthopaedic Surgery, St Vincent’s Hospital Melbourne, Melbourne, Victoria, Australia; Australian Orthopaedic Association National Joint Replacement Registry (AOANJRR), South Australian Health and Medical Research Institute (SAHMRI), Adelaide, South Australia, Australia; School of Medicine, The University of Adelaide, Adelaide, South Australia, Australia; Australian Orthopaedic Association National Joint Replacement Registry (AOANJRR), South Australian Health and Medical Research Institute (SAHMRI), Adelaide, South Australia, Australia; Queensland University of Technology, Brisbane, Queensland, Australia; Australian Orthopaedic Association National Joint Replacement Registry (AOANJRR), South Australian Health and Medical Research Institute (SAHMRI), Adelaide, South Australia, Australia; Menzies Health Institute, Griffith University, Queensland, Australia; Australian Orthopaedic Association National Joint Replacement Registry (AOANJRR), South Australian Health and Medical Research Institute (SAHMRI), Adelaide, South Australia, Australia; Department of Orthopaedic Surgery, St Vincent’s Hospital Melbourne, Melbourne, Victoria, Australia; Australian Orthopaedic Association National Joint Replacement Registry (AOANJRR), South Australian Health and Medical Research Institute (SAHMRI), Adelaide, South Australia, Australia; Australian Orthopaedic Association National Joint Replacement Registry (AOANJRR), South Australian Health and Medical Research Institute (SAHMRI), Adelaide, South Australia, Australia; Australian Orthopaedic Association National Joint Replacement Registry (AOANJRR), South Australian Health and Medical Research Institute (SAHMRI), Adelaide, South Australia, Australia; Australian Orthopaedic Association National Joint Replacement Registry (AOANJRR), South Australian Health and Medical Research Institute (SAHMRI), Adelaide, South Australia, Australia; South Australian Health and Medical Research Institute (SAHMRI), Adelaide, South Australia, Australia; Australian Orthopaedic Association National Joint Replacement Registry (AOANJRR), South Australian Health and Medical Research Institute (SAHMRI), Adelaide, South Australia, Australia; Australian Orthopaedic Association National Joint Replacement Registry (AOANJRR), South Australian Health and Medical Research Institute (SAHMRI), Adelaide, South Australia, Australia; Australian National University, Canberra, Australian Capital Territory, Australia

**Keywords:** AOANJRR, Australian Orthopaedic Association Joint Replacement Registry, cohort profile, data resource profile, descriptive summary, orthopaedic surgery, arthroplasty, total hip replacement, total knee replacement, total shoulder replacement

Key Features

- The Australian Orthopaedic Association National Joint Replacement Registry (AOANJRR) is an Australian clinical quality registry that was established to define, maintain, and improve the quality of care for individuals undergoing joint-replacement surgery.
- The AOANJRR has collected data for 99.0% of the primary and revision joint-replacement procedures performed in Australia since 1 September 1999. To date, the Registry has collected data for >2 million procedures and reports 20-year outcomes.
- The AOANJRR cohort is a continuously updated dataset. Patient, implant, and procedural data are collected, with the recent addition of patient-reported outcome measures.
- The primary outcome measure is revision surgery, defined as the removal, replacement, or addition of any device component. Linkage of the AOANJRR to the Australian National Death Index allows the reporting of patient mortality.
- Interested collaborators can contact the AOANJRR team at https://aoanjrr.sahmri.com/.

## Data resource basics

The Australian Orthopaedic Association National Joint Replacement Registry (AOANJRR) is a national healthcare initiative with the primary objectives of defining, maintaining, and improving the quality of care for individuals undergoing joint-replacement surgery.^1^ First suggested in 1993 by the Australian Orthopaedic Association (AOA), the establishment of the AOANJRR was a consequence of limited understanding of joint-replacement outcomes.^2^^,^^3^ Inspired by the early success of registries in Sweden (since 1979), Finland (since 1980), and Norway (since 1987), the concept of a national joint registry in Australia was proposed to the Commonwealth Government in 1996 and approved for funding in 1998.^4–6^ The first data collection occurred in September 1999 as a pilot cohort, and full national implementation had occurred by January 2003. As of June 2024, the AOANJRR collects data on joint replacements involving the hip, knee, shoulder, elbow, wrist, ankle, and spinal disc, as well as knee osteotomy procedures for arthritis.

The AOANJRR (also termed ‘the Registry’ in this manuscript) remains an independent entity within the AOA, but is financially supported through funding from the Commonwealth Government.^7^ Before 2009, the AOANJRR received funding through grants from the Federal Department of Health and Aged Care (DoHAC) budget. However, in 2009, federal legislation [Private Health Insurance (National Joint Replacement Register Levy) Act 2009] allowed the maintenance costs of the AOANJRR to be partly recuperated through levies imposed on the orthopaedic industry and other commercial stakeholders.^8^ Furthermore, the data collected by the AOANJRR are protected by the Commonwealth Government under the auspices of a Federal Quality Assurance Activity.^1^

Since its inception, the AOANJRR has amassed data on >2 million procedures, accumulated over 24 years of continuous follow-up data, and is currently managed by a dedicated team of >50 clinicians, researchers, and administrators. Furthermore, due to the designation of the AOANJRR as a Federal Quality Assurance Activity, the Registry receives data from all hospitals in Australia that are performing joint replacement and captures >99% of all joint procedures performed in Australia.^1^  Table 1 provides demographic data for all 2 157 682 joint replacements recorded in the AOANJRR to date. Table 2 provides demographic data for all 1 726 668 primary total hip, knee, and shoulder replacements, performed for all diagnoses, currently recorded in the Registry. Table 3 provides demographic data for all 188 675 revision hip, knee, and shoulder replacements, performed for all diagnoses, recorded in the AOANJRR to date.

**Table 1.** Age and sex demographics for all joint replacements.

	Number		Age (years)
Joint replacement	Total	Female (%)	Mean ± SD (range)
Hip	910 050	510 041 (56.0)	69.9 ± 12.6 (5–108)
Knee	1 125 214	617 128 (54.8)	68.3 ± 9.4 (8–107)
Shoulder	95 786	56 707 (59.2)	71.4 ± 9.6 (12–103)
Elbow	7 719	4 919 (63.7)	59.9 ± 16.2 (12–102)
Wrist	1 074	516 (48.0)	62.6 ± 13.7 (18–90)
Ankle	5 361	2 041 (38.1)	67.4 ± 9.1 (20–94)
Spinal Disc	12 478	5 471 (43.8)	45.7 ± 10.8 (15–99)
Total	2 157 682		

This table includes data for all primary partial, primary total, and revision joint replacements, performed for all diagnoses. Data for spinal disc replacement include primary procedures only. Data obtained from Tables SD1, SD31, and SD57 in the Demographics of Hip, Knee and Shoulder Arthroplasty 2024 Supplementary Report; Tables EG4 and WG3 from the Demographics and Outcomes of Elbow and Wrist Arthroplasty 2024 Supplementary Report; Table A3 from the Demographics and Outcome of Ankle Arthroplasty 2024 Supplementary Report; and Table SD5 from the Demographics of Spinal Disc Arthroplasty 2024 Supplementary Report.^12^

**Table 2.** Age and sex demographics for all primary total hip, knee, and shoulder replacements.

	Number			Age (years)
Joint replacement	Total	Female (%)		Mean ± SD (range)
Hip	691 376		372 508 (53.9)	67.4 ± 11.7 (11–108)
Knee	956 175		534 205 (55.9)	68.5 ± 9.2 (8–107)
Shoulder	79 117		47 463 (60.0)	72.1 ± 8.7 (12–103)
Total	1 726 668			

This table includes data for all primary total hip, knee, and shoulder replacements, performed for all diagnoses. Data obtained from Tables SD20, SD52, and SD76 in the Demographics of Hip, Knee and Shoulder Arthroplasty 2024 Supplementary Report.^12^

**Table 3.** Age and sex demographics for all revision hip, knee, and shoulder replacements.

	Number		Age (years)
Joint replacement	Total	Female (%)	Mean ± SD (range)
Hip	91 456	48 710 (53.3)	71.0 ± 12.1 (11–104)
Knee	88 657	45 046 (50.8)	69.1 ± 10.3 (10–101)
Shoulder	8 562	4 472 (52.2)	70.0 ± 10.3 (15–98)
Total	188 675		

This table includes data for all revision hip, knee, and shoulder replacements, performed for all diagnoses. Data obtained from Tables SD 28, SD54, and SD88 in the Demographics of Hip, Knee and Shoulder Arthroplasty 2024 Supplementary Report.^12^

## Data collected

### AOANJRR data sources

The majority of AOANJRR data are collected by using data-collection forms, available on the AOANJRR website at https://aoanjrr.sahmri.com/. These forms collect the three core domains of data—patient, surgical, and implant details. As a Federal Quality Assurance Activity, patient consent is not required for data collection; however, patients have the option to opt out if they choose not to participate, by contacting the Registry via a free-call telephone number. Further details about these domains of data are available in the Supplementary Materials and the AOANJRR Data Set Specification, available online.^9^

### Data-collection processes

Data-collection forms are completed by hospital staff at the time of surgery. These physical forms are mailed to the AOANJRR on a monthly basis.^1^ Upon receiving these forms, a specialized data-entry team inputs the information into the AOANJRR database. To ensure accuracy and address any immediate discrepancies, data managers are available on-site during the data-entry process. The data-entry system is equipped with a predictive-text feature to minimize typographical errors. While the AOANJRR has established protocols for the transition to electronic data submission, this method has not yet been adopted by any participating hospitals at the time of publication.

### Key outcome measures

The AOANJRR primarily tracks joint-replacement trends, revision rates, reasons for revision, and patient mortality. A revision is defined as the removal, replacement, or addition of any device component and categorized as major (involving primary components such as the acetabulum, femur, or tibia) or minor (e.g. revisions of the femoral head, acetabular liner, or patella). Surgeons can record multiple reasons for revision, with a hierarchical system used for reporting the main cause. Mortality information is obtained by matching all procedures with the National Death Index—a national mortality database maintained by the Australian Institute of Health and Welfare (AIHW). Twice a year, the AOANJRR provides an extract of demographic data to the AIHW for data linking to determine morality rates.

In 2018, the AOANJRR launched an 18-month pilot for collecting patient-reported outcome measures (PROMs) prior to a national rollout in 2020. PROMs are captured by using RAPID (Real time Automated Platform for Integrated Data capture), an electronic platform, and patients are reminded via text message to complete questionnaires before surgery and 6 months afterward.^10^ Completion rates during the pilot ranged from 50% to 70%, with ongoing efforts to address responder bias due to missing data.^11^

### Data validation

The AOANJRR ensures data integrity through a validation process that cross-references information from hospitals and health departments, addressing discrepancies in patient identifiers, procedure codes, and dates.^3^ Errors are resolved by consulting with hospital coordinators and verifying data against state and territory records. This rigorous process achieves a 99% data-capture rate for hip, knee, and shoulder joint-replacement procedures in Australia.^1^ Further details are provided in the Supplementary Materials.

### Data evolution

The AOANJRR is consistently expanding, re-evaluating, and updating its dataset (Table 4). Data collection for shoulder, ankle, elbow, wrist, and spinal disc replacements commenced nationally in 2007. Based on evolving practices in joint-replacement surgery, the AOANJRR has included additional variables in its dataset on several occasions. Technology assistance [e.g. computer navigation or image-derived instrumentation (2009)] in total hip (THR) and knee replacement (TKR) was included in 2009, American Society of Anaesthesiologists score in 2012, body mass index (BMI) in 2015, and surgical approach for THR in 2015.

**Table 4.** Timeline of key events in the establishment and development of the AOANJRR.

Year	Key event
1999	Hip and knee replacement data collection commences
1999	Declared a Federal Quality Assurance Activity
2003	Full national hip and knee replacement data collection achieved
2003	Computer navigation data collection commences
2004	Identification of prosthesis outliers established
2004	Shoulder replacement data collection commences
2005	Elbow replacement data collection commences
2006	Ankle and wrist replacement data collection commences
2007	Full national shoulder, elbow, wrist, ankle, and spinal disc replacement data collection
2009	Permanently funded by the Commonwealth Government
2009	IDI data collection commences
2012	ASA score data collection commences
2015	BMI data collection commences
2015	Robotic assistance for partial knee replacement data collection commences
2015	Surgical approach for total hip replacement data collection commences
2016	Robotic assistance for total knee replacement data collection commences
2018	PROMs pilot programme commences
2020	National rollout for PROMs data collection commences

ASA, American Society of Anaesthesiologists; BMI, body mass index; IDI, image-derived instrumentation; PROMs, patient-reported outcome measures.

## Data resource use

### Hip, knee, and shoulder joint-replacement Annual Reports

The AOANJRR publishes an Annual Report detailing trends and outcomes in joint-replacement surgery, analysed by statisticians and reviewed by Clinical Directors, AOA fellows, and subspecialty members. After AOA Board approval, the report is made publicly available on the AOANJRR website. A lay version is provided for patients. Supplementary reports on specific topics such as PROMs, demographics, and partial joint replacements are also published annually and accessible online.^12^

### Identification of higher-than-anticipated revision rates of prostheses

The identification of prostheses with higher-than-anticipated revision rates (HTARR) by the AOANJRR is a core function of the Registry and is a multistage process. In Stage 1, automated analysis flags prostheses with revision rates exceeding twice the class median by using Poisson probabilities (*P* < 0.05) and other thresholds. Prostheses may be assessed individually or in combination, depending on the performance variability. In Stage 2, AOANJRR Clinical Directors, statisticians, and data managers conduct detailed investigations, adjusting for confounding factors and calculating hazard ratios. If differences remain significant, then the prosthesis or combination moves to Stage 3, in which an independent panel of orthopaedic surgeons reviews the findings to determine inclusion in the Annual Report. Regulatory representatives may also participate, underscoring the importance of the process in ensuring prosthesis safety and performance.

### Surgeon and hospital annual reports

The AOANJRR prepares confidential surgeon reports and hospital reports, allowing comparison against national benchmarks while accounting for case-mix variability. Surgeons can access less-detailed data via a secure portal and hospital reports anonymize surgeon identities, ensuring privacy in cases with multiple surgeons and sufficient procedure volume.

### Collaboration with regulatory authorities and industry

The AOANJRR collaborates with Australian regulatory bodies such as the DoHAC and Therapeutic Goods Administration, providing real-time data on device revision rates to support safety monitoring and regulatory oversight. Through secure portals, both regulators and industry partners can access daily updates on prosthesis performance, with manufacturers benefiting from the Automated Industry Reporting System for customized, frequent revision data.

### Collaboration with other registries

The AOANJRR is an active member of the International Society of Arthroplasty Registries [13]. This collaboration strengthens the consistency of terminology and analytical techniques, facilitates the international benchmarking of prosthetic outcomes, and enables international collaboration on research projects. Such collaboration is particularly useful when examining research questions that require large datasets to investigate unusual diagnoses or prosthesis outcomes.

### Research

Despite the original purpose of the AOANJRR to act as a quality-assurance activity, the quality and completeness of the Registry dataset mean that it is an excellent resource for orthopaedic research, particularly in understanding joint-replacement outcomes. Methodical examination of Registry data allows the discernment of patterns, the frequency of complications, and the long-term efficacy of various implants. Such analyses are crucial in establishing the superior performance of certain prostheses and in determining the efficacy of surgical techniques.

The longitudinal monitoring capabilities of the AOANJRR are equally significant for the post-market evaluation of implants. This aspect of the Registry is fundamental in recognizing implants that exhibit elevated rates of revision, thus facilitating prompt remedial action.

The AOANJRR also supports epidemiological investigations by providing insights into how demographic variables such as age, sex, and BMI interact with surgical outcomes. Furthermore, the recent use of the Registry to conduct Registry Nested Clinical Studies (RNCS) has allowed a more consistent approach to following up patient outcomes during participation in clinical trials. This is discussed in further detail below.

Throughout the 21 years of continuous data collection, the AOANJRR has achieved some significant and practice-changing outcomes. Through the HTARR implant identification process, the AOANJRR was the first registry to identify high revision rates associated with the ASR (Articular Surface Replacement, Depuy, USA) conventional THR implant and subsequently the ASR hip resurfacing implant.^14^ Through the same process, additional implants and design categories have been identified as having a high revision risk. Examples include modular neck femoral implants and non-cross-linked polyethylene (NXLPE) acetabular components.^1^^,^^15^ Due to the longitudinal nature of the AOANJRR data, changes in practice following landmark registry studies are also captured. These include an increase in the use of highly cross-linked polyethylene (XPLE) in THR, an increase in patella resurfacing in TKR, and an increase in the proportion of reverse total shoulder replacement (rTSR) In recent times, landmark papers on technology-assisted surgery have been published by using AOANJRR data. An example of this is the identification of computer navigation improving the revision rates for patients <65 years of age undergoing TKR.^16^

The AOANJRR has contributed to many peer-reviewed research articles, as well as several RNCS. Some examples are provided below. RNCS represent a significant advancement in the ability to conduct randomized–controlled trials (RCTs). They leverage existing cohorts or databases such as the AOANJRR for efficiency and practicality. This approach significantly reduces the resources required compared with conventional trials, allowing the recruitment of large, representative samples that closely reflect real-world clinical practices.

The CRISTAL (cluster-randomized, crossover, non-inferiority trial of aspirin compared with low molecular weight heparin for venous thromboembolism prophylaxis in hip or knee arthroplasty, a registry nested study) trial was the first large RNCS conducted through the AOANJRR.^17^ Despite the CRISTAL trial recruiting >15 000 participants, the overall cost of the study was <$1 million AUD. In comparison, most large-scale surgical RCTs cost >$5 million AUD to conduct.^18^ This exemplifies the potential cost savings attributed to the streamlined processes of RNCS. Other RNCS are currently in progress, including the RASKAL (robotic-assisted surgery and kinematic alignment in total knee arthroplasty) and DISTINCT (dual-mobility versus conventional total hip arthroplasty in femoral neck fracture) trials, which are expected to be completed by 2025 and 2028, respectively.^19^^,^^20^

### Future directions

As the AOANJRR dataset continues to grow, it will allow the completion of more detailed scientific analyses. This will allow the Registry to better deliver its core aim to improve joint-replacement outcomes both for individual patients and the healthcare system by delivering robust data that will engender practice change. Data collection in a cost-effective manner is paramount and the AOANJRR is actively examining the latest methods for data capture, processing, and storage. Linkage of multiple datasets in concert with machine-learning techniques will be integral to the AOANJRR achieving its aims. Whilst technological advances will improve the AOANJRR, its ultimate aim is to improve the communication of clinically relevant data in a clear and timely fashion to its various stakeholders so that clinical care can be optimized as rapidly as possible.

## Strengths and weaknesses

### Strengths

The AOANJRR has several key strengths. One of its most significant is the comprehensive and high-quality national data that it provides. This extensive coverage enables detailed monitoring of revision rates, implant performance, and patient outcomes across multiple joint types. In addition, the Registry’s data-validation processes ensure a high level of accuracy, while its ability to track long-term outcomes over more than two decades provides invaluable insights into the efficacy and safety of various prostheses and surgical techniques. Furthermore, the AOANJRR actively collaborates with international registries and regulatory authorities, thereby improving the impact of its findings and supporting global improvements in joint-replacement care. Its research contributions, such as identifying high-revision-risk implants and facilitating cost-effective registry-based clinical studies, make the AOANJRR an essential tool for improving patient care and advancing the field of orthopaedics.

### Weaknesses

Despite the strengths of the AOANJRR, there are also limitations to consider. In national joint-replacement registries such as the AOANJRR, minimum datasets are commonly used to ensure comprehensive data collection.^21^ However, this approach often falls short in providing in-depth analysis concerning outcomes, confounders, and risk factors. The primary outcome of the AOANJRR is revision surgery, which means that symptomatic patients who do not undergo revision are not identified. Incorporating PROMs data may offer a broader understanding of these cases.^22^ However, this is associated with challenges of its own. There are multiple PROMs tools currently in use, each with its own advantages and disadvantages.^23^ Quality-of-life (QoL) scales such as the EQ-5D-5L can capture an assessment of overall health but may not be sensitive to small differences in joint function. Conversely, joint-specific PROM tools such as the Oxford Knee Score capture the symptoms of patients’ joints in detail, but often neglect to consider the wider QoL experiences.^24^ Furthermore, the issue with missing data in PROMs remains a concern for the interpretability of this type of data.^25^

It is important to acknowledge that, while the AOANJRR promotes the value of observational data, it also recognizes the risk of misinterpretation. To mitigate this, the Academic Editorial Advisory Panel (AEAP) reviews all publications based on Registry data, prior to submission. The observational nature of the AOANJRR (like any registry dataset) has significant limitations to drawing inferences of causality. Therefore, interpreting, disseminating, and implementing the findings of the AOANJRR require careful consideration. The principles of sound clinical judgement remain applicable to all findings reported by the AOANJRR.

## Data resource access

The AOANJRR adheres to strict legal and ethical standards that limit data access primarily to aggregated summaries. Interested researchers are invited to engage with the Registry through a structured ad hoc data-request process.^1^ This entails submitting a comprehensive application that outlines the research objectives, methodology, and expected outcomes, with the inclusion of an AOA Fellow on the requesting team. These applications undergo evaluation by the AOANJRR Clinical Directors, who consider the scientific merit, practicality, and ethical implications of the data request, as well as any potential overlap with ongoing or previous research. Approved requests are then analysed by dedicated statisticians, who compile and provide the summarized data to the researchers. Before any dissemination of data, the final output must receive approval from the AOANJRR Clinical Directors, and publications must also be approved by the AEAP to ensure that the information is communicated accurately.

Separate ad hoc request processes exist for hospitals, government organizations, and implant companies to access AOANJRR data. Applications are assessed and implemented via a similar process to that for research requests. Interested collaborators should contact the AOANJRR Publications Manager (datarequests@aoanjrr.org.au).

## Ethics approval

Ethical committee approval was not required for the study. The AOANJRR is approved by the Commonwealth of Australia as a Federal Quality Assurance Activity (F2022L00986) Part VC of the Health Insurance Act 1973 and Part 10 of the Health Insurance Regulations 2018.

## Supplementary Material

dyaf078_Supplementary_Data

## Data Availability

Aggregate data underlying this article will be shared on reasonable request to the corresponding author. For interested collaborators, please contact the AOANJRR Publications Manager, Dr Sophie Corfield (datarequests@aoanjrr.org.au).
