# Supplementary material for: Data Resource Profile: The Australian Orthopaedic Association National Joint Replacement Registry (AOANJRR)
Source: Int J Epidemiol. 2025 Jun 16;54(4):dyaf078. doi: 10.1093/ije/dyaf078 (PMC12199916; doi:10.1093/ije/dyaf078)
Supplement: dyaf078_Supplementary_Data [file dyaf078_supplementary_data.docx]

**SUPPLEMENTARY MATERIAL**

*Specific Data Variables*

Aligning with the primary objectives of the AOANJRR, data collection focuses on three broad categories – patient, surgical, and implant details.^1^ This is commonly known as the minimum data set. While the minimum data set has remained consistent since its inception, minor adjustments have occurred over time.^2^ Furthermore, some variables are only captured for specific procedures. Detailed variable specifications can be accessed through the data dictionary presented in the Supplementary Materials.The most up-to-date version of the data dictionary is available on the AOANJRR website (<https://aoanjrr.sahmri.com>).^3^

Patient details include personal identifiers, postcode, birth date, sex, surgical date, the hospital where the surgery was conducted, the American Society of Anaesthesiologists physical status classification system (ASA) score, and body mass index (BMI). Surgical details include the identity code of the operating surgeon, the type of procedure, the affected side of the body, the technical specifics of the surgical method including the approach used for total hip replacement (THR), glenoid morphology for shoulder joint replacement, mode of implant fixation, and the use of technological assistance, such as computer navigation, patient-specific instrumentation, and robotic-assisted surgery. Implant details include detailed records such as the manufacturer, prosthesis type, implant model, catalogue and series numbers, the material of the implant and components, and the use of bone grafts or supplementary fixation.

*Data Validation*

The AOANJRR ensures the integrity of its data through a meticulous validation process that cross-references the information received from both public and private hospitals with that of State and Territory health departments.^4^ This validation process identifies:

1. Procedures reported to the health departments that are also documented in the Registry.
2. Procedures where the State or Territory has a record of a procedure that the Registry has not been notified by the hospitals.
3. Procedures where both the Registry and health departments have identical records (‘exact matches’).
4. Procedures that are partially consistent between the Registry and health departments but require further corroboration from the hospital for complete verification.

In the initial stages of data validation, the Registry conducts a verification of hospital and patient identifiers, followed by a verification of the corresponding procedure codes and the dates of admission.^5^ Discrepancies may emerge at any point in this process. These include errors in the identification of patients, the coding of procedures, or in the recording of data by the hospitals, health authorities, or the Registry. Discrepancies are addressed according to their specific details. For example, when there is a discrepancy in the type of procedure recorded by the health department and the Registry, the Registry's account is typically upheld as it contains detailed information about the implanted devices.

Uncertainties with the collected data are also corrected by direct contact with each hospital’s nominated Registry co-ordinator. In addition, the AOANJRR uses data available from State and Territory records to verify discrepancies between Registry and hospital data.^6^ Following these data validation processes, the Registry succeeds in compiling a dataset that is near-complete, achieving a 99.0% rate of data capture for all hip, knee, and shoulder joint replacement procedures in Australia.^1^

*Key Outcome Measures*

In addition to tracking the volume and trends in joint replacement procedures and techniques, the AOANJRR also presents key outcomes such as revision rates, indications for revisions, patient mortality (linked with the Australian National Death Index [NDI]), and more recently, patient reported outcome measures (PROMs). A revision procedure is defined as removal, replacement, or addition of any device component.^11^ These are further sub-categorised as major and minor revisions. A major revision involves either acetabular or femoral or both components (for hips); femoral or tibial or both components, but not the patella (for knees); and humeral or glenoid or both components (for shoulders). Minor revisions typically involve femoral head, acetabular liner, tibial insert, and patella revisions.^1^ The reasons for revision are categorised and recorded using pre-defined options, although surgeons also have the flexibility to provide additional free-text information. Multiple reasons for revision can be recorded. The AOANJRR then categorises these reasons into specific diagnoses. A hierarchical classification system is used to determine the main reason for surgery which is used for reporting purposes.

In July 2018, the AOANJRR commenced an 18-month PROMs collection pilot project, prior to national implementation in 2020. PROMs are captured using a variety of joint specific and global health instruments.^12^ Data is recorded using an electronic PROM capture platform known as *RAPID* (Real time Automated Platform for Integrated Data capture), which was specifically designed for the needs of the AOANJRR.^9^ Short Message Service (SMS) text messages with a reminder to complete PROM questionnaires are sent to patients via the *RAPID* platform before surgery and 6-months after the surgery. During the pilot project, completion rate of PROMs was estimated to be between 50-70% for both pre-surgery and 6-month responses.^10^ Ongoing efforts are in place to improve the completion rate of PROMs data, noting the concerns with responder bias associated with missing data.^13^

*Legality and Ethics*

The AOANJRR is approved by the Commonwealth of Australia as a Federal Quality Assurance Activity (F2022L00986) Part VC of the Health Insurance Act 1973 (HIA) and Part 10 of the Health Insurance Regulations 2018.^7^ This was first designated in 2009 and was subsequently re-updated in 2015 and 2022. Designation as a Federal Quality Assurance Activity ensures that the AOANJRR is provided legal benefits and protections. These include a reduction in disclosure risk and protections from civil liabilities for participants (both patients and surgeons).^8^ The consequence of these protections is to encourage widespread participation in the AOANJRR to ensure the outcomes reported are of the highest standards for healthcare improvement. Patients may opt-out of participation in the AOANJRR, but this is rarely chosen. Surgeons can decline to provide their surgeon code on the data collection forms, however, the procedures are still recorded and followed-up.

Data collection, management, and analysis is performed by an independent entity (currently the South Australian Health and Medical Research Institute [SAHMRI]) contracted by the AOANJRR. As a third-party, this minimises any biases associated with the AOANJRR performing these tasks within the organisation.

*Funding*

Before 2009, the AOANJRR received funding through grants from the Federal Department of Health and Aged Care budget. However, in 2009, the funding model shifted to align with the Australian Government Cost Recovery Policy.^9^ Under this model, medical device companies are levied for specific implant components listed on the Prescribed List, managed by the Department of Health and Aged Care.^10^ This list outlines prostheses/implants that private health insurance companies must reimburse for privately insured patients. Funds are allocated to the AOA through a budget process and funding agreement between the AOA and the Department of Health and Aged Care.

*Hip, Knee, and Shoulder Joint Replacement Annual Reports*

The AOANJRR publishes a comprehensive Annual Report detailing trends and outcomes in joint replacement surgery, with data analysed by statisticians and reviewed by AOANJRR Clinical Directors. The final draft is assessed at a workshop for AOA Fellows and subspecialty members before AOA Board approval and public release. This report, available on the AOANJRR website ^1^, keeps surgeons, patients, insurers, hospitals, industry partners, regulators, and government bodies informed, aligning with AOANJRR's objective to improve joint replacement care standards. A lay version is also available for patients. Additionally, the AOANJRR publishes annual Supplementary Reports on specific aspects of joint replacement, such as PROMs, patient demographics, mortality, or partial joint replacement, which are also accessible on their website.

*Identification of Higher Than Anticipated Revision Rate prostheses*

Identification of prostheses with higher than anticipated revision rates (HTARR) involves a multi-stage process conducted by the AOANJRR. In Stage 1, an automated analysis is performed using predetermined criteria. This includes identifying prostheses with revision rates exceeding twice the combined median for their class, with a significant Poisson probability (p<0.05), and meeting certain procedural thresholds or exhibiting high revision proportions. The analysis considers both individual prostheses and combinations thereof, recognising that performance may vary based on the combination used. Prostheses identified in specific combinations proceed to Stage 2, while those identified across multiple combinations or uniformly proceed individually to Stage 2. The second stage involves a detailed investigation by AOANJRR Clinical Directors, statisticians, and SAHMRI staff, examining potential confounding factors and calculating adjusted hazard ratios. Previous reports are also re-analysed at this stage. If significant differences persist compared to other prostheses in the same class, the prosthesis or combination advances to Stage 3. Stage 3 entails a final review by an independent panel of orthopaedic surgeons from subspecialty societies, who meet with Registry staff to assess Stage 2 findings. The panel ultimately determines which prostheses will be included in the Annual Report. Representatives from regulatory bodies may also attend the review workshop.

*Surgeon and Hospital Annual Reports*

The AOANJRR also prepares confidential individualised surgeon reports annually and provides hospital reports upon request. These reports allow for comparative analysis against national benchmarks and reflective analysis of outcomes and practices. They are not definitive performance indicators due to case mix variability. Surgeons with an AOA code can access less detailed data anytime via a secure portal, showing procedure numbers, revision rates, and diagnoses. To protect surgeon privacy, hospital reports anonymise individual surgeons, including analyses only if at least three surgeons have used an implant, with a minimum of 50 procedures and 4 revisions performed.

*Collaboration with Regulatory Authorities and Industry*

The AOANJRR fosters a constructive relationship with Australian regulatory authorities, including the Department of Health and Aged Care (DoHAC) and the Therapeutic Goods Administration (TGA), which regulate and monitor the safety, efficacy, quality, and clinical performance of therapeutic goods in Australia. This collaboration is epitomised by dedicated portals for both the DoHAC and the TGA to access real-time data on device revision rates, providing actionable insights, especially considering recent regulatory amendments.^14^ This strategic partnership underscores a shared commitment to safeguarding public health by ensuring continuous surveillance of medical devices. Similarly, the AOANJRR collaborates with industry through a secure web portal providing real-time information on prosthesis performance, updated daily but unverified, allowing for detailed monitoring. The AOANJRR has also developed the Automated Industry Reporting System (AIRS), enabling manufacturers to access customised, frequent revision data on their implants, with automated reports available on specific components or surgical techniques upon request.

*Collaboration with Other Registries*

The AOANJRR is an active member of the International Society of Arthroplasty Registries (ISAR). There are 35 registries who are members of ISAR.^15^ This collaboration strengthens consistency of terminology and analysis techniques, facilitates international benchmarking of prosthetic outcomes, and enables international collaboration on research projects. Such collaboration is particularly useful when examining research questions that require large datasets to attain robust outcomes such as studies of post-operative infection with the use of antibiotic loaded bone cement.

*Data Custodianship*

The AOANJRR is overseen by several committees and operated on a day-to-day basis by a dedicated team of full-time staff. Oversight begins with the AOA Registry Committee, which reports to the Board of Directors of the AOA. This committee comprises orthopaedic surgeons from each state and sub-specialty joint replacement group. Additionally, a team of Clinical Directors, appointed by the AOA Board, provides strategic and clinical guidance, ensuring effective collaboration with hospitals, government departments, and international counterparts. A parallel structure exists for operational management. The Registry's day-to-day functions, strategic implementations, data reporting, research activities, and preparation of reports are managed by an operational team, including the Registry Executive Manager, along with various coordinators and officers.

**REFERENCES**

1. Smith PN, Australian Orthopaedic Association National Joint Replacement Registry, Gill DRJ, et al. Hip, knee & shoulder arthroplasty: 2023 Annual Report [Internet]. Australian Orthopaedic Association; 2023 Oct. Available from: http://dx.doi.org/10.25310/ywqz9375

2. Aoanjrr S. Data Set Specification [Internet]. [cited 2023 Nov 19]. Available from: https://aoanjrr.sahmri.com/documents/10180/723197/AOANJRR+Data+Dictionary/7eb500dd-d999-0b3d-43ca-abf917596476

3. [Cited 2024 Apr 7]. Available from: https://aoanjrr.sahmri.com/documents/10180/723197/AOANJRR+Data+Dictionary.pdf/7eb500dd-d999-0b3d-43ca-abf917596476?t=1704849943528

4. Steiger RN de, Graves SE. Orthopaedic registries: the Australian experience. *EFORT Open Rev* [Internet]. Bioscientifica; 2019 Jun;**4**(6):409–415. Available from: http://dx.doi.org/10.1302/2058-5241.4.180071

5. Porter M, Rolfson O, Steiger R de. International registries: U.k. national joint registry, Nordic registries, and Australian orthopaedic association national joint replacement registry (AOANJRR). *J Bone Joint Surg Am* [Internet]. 2022 Oct 19;**104**(Suppl 3):23–27. Available from: http://dx.doi.org/10.2106/JBJS.22.00561

6. Harris IA, Cashman K, Lorimer M, et al. Are responders to patient health surveys representative of those invited to participate? An analysis of the Patient-Reported Outcome Measures Pilot from the Australian Orthopaedic Association National Joint Replacement Registry. *PLoS One* [Internet]. Public Library of Science (PLoS); 2021 Jul 2;**16**(7):e0254196. Available from: http://dx.doi.org/10.1371/journal.pone.0254196

7. [Cited 2023 Nov 19]. Available from: http://www5.austlii.edu.au/au/legis/cth/consol_act/hia1973164/

8. Ahern S, Hopper I, Loh E. Qualified privilege legislation to support clinician quality assurance: balancing professional and public interests. *Med J Aust* [Internet]. AMPCo; 2019 May;**210**(8):343-346.e1. Available from: http://dx.doi.org/10.5694/mja2.50124

9. [Cited 2024 Mar 20]. Available from: https://www.finance.gov.au/government/managing-commonwealth-resources/implementing-charging-framework-rmg-302/australian-government-cost-recovery-policy#:~:text=Cost

10. [Cited 2024 Mar 20]. Available from: https://www.health.gov.au/topics/private-health-insurance/the-prostheses-list

11. McEwan J. A history of therapeutic goods regulation in Australia. tga.gov.au; 2007; Available from: https://www.tga.gov.au/sites/default/files/history-tg-regulation.pdf

12. [Cited 2023 Nov 16]. Available from: https://www.tga.gov.au/how-we-regulate/supply-therapeutic-good/supply-medical-device/medical-devices-reforms/medical-device-reforms-regulatory-changes

13. Hasan SS, Kow CS, Dawoud D, Mohamed O, Baines D, Babar Z-U-D. Pharmaceutical Policy Reforms to Regulate Drug Prices in the Asia Pacific Region: The Case of Australia, China, India, Malaysia, New Zealand, and South Korea. *Value Health Reg Issues* [Internet]. Elsevier; 2019 May;**18**:18–23. Available from: http://dx.doi.org/10.1016/j.vhri.2018.08.007

14. Kim H, Byrnes J, Goodall S, ISPOR Australia Chapter executive committee. Health Technology Assessment in Australia: The Pharmaceutical Benefits Advisory Committee and Medical Services Advisory Committee. *Value Health Reg Issues* [Internet]. Elsevier; 2021 May;**24**:6–11. Available from: http://dx.doi.org/10.1016/j.vhri.2020.09.001

15. Malchau H, Garellick G, Berry D, et al. Arthroplasty implant registries over the past five decades: Development, current, and future impact. *J Orthop Res* [Internet]. 2018 Sep;**36**(9):2319–2330. Available from: http://dx.doi.org/10.1002/jor.24014
